# Successful and rapid response of speech bulb reduction program combined with speech therapy in velopharyngeal dysfunction: a case report

**DOI:** 10.1186/s40902-015-0022-4

**Published:** 2015-08-06

**Authors:** Yu-Jeong Shin, Seung-O Ko

**Affiliations:** 1 Department of Language Therapy, Howon University, Howondae-3gil, Leempi-myun, Gunsan, 573-718 Jeollabuk-do Korea; 2 Department of Oral and Maxillofacial Surgery, School of Dentistry, Research Institute of Clinical Medicine of Chonbuk National University-Biomedical Research Institute of Chonbuk National University Hospital, Geonjiro-20 Deokjin-gu, Jeonju, 561-712 Jeollabuk-do Korea

**Keywords:** Velopharyngeal dysfunction, VPD, Velopharyngeal insufficiency, Velopharyngeal incompetence, VPI, Speech aid prosthesis, Speech therapy, Speech bulb

## Abstract

Velopharyngeal dysfunction in cleft palate patients following the primary palate repair may result in nasal air emission, hypernasality, articulation disorder and poor intelligibility of speech. Among conservative treatment methods, speech aid prosthesis combined with speech therapy is widely used method. However because of its long time of treatment more than a year and low predictability, some clinicians prefer a surgical intervention. Thus, the purpose of this report was to increase an attention on the effectiveness of speech aid prosthesis by introducing a case that was successfully treated. In this clinical report, speech bulb reduction program with intensive speech therapy was applied for a patient with velopharyngeal dysfunction and it was rapidly treated by 5months which was unusually short period for speech aid therapy. Furthermore, advantages of pre-operative speech aid therapy were discussed.

## Background

Velopharyngeal dysfunction (VPD) is a term describing an inappropriate function of velopharyngeal (VP) port which consists of lateral and posterior pharyngeal walls and soft palate. This muscular valve can control the air passage between oro- and nasopharynx. When the proper closure cannot be performed, liquid regurgitation during swallowing, nasal air emission, hyper-nasality and poorly intelligible speech may occur [1]. Furthermore, this physical disability usually causes psychological stress on the patients with VPD, especially during childhood [2].

The impairment of velopharyngeal function can be attributed to structural causes, neurologic causes and speech mislearning [3]. Even though there is sufficient soft tissue to close the VP port with normal anatomical structure, velopharyngeal function can be incompetence (velopharyngeal incompetence; VPI) due to neuromuscular disorders: cerebral palsy, myotonic dystrophy, cerebral vascular accidents, etc. On the other hand, soft tissue deficiency for closing VP port, surgical removal or congenital loss of normal structure separating the nasal and oral cavity can lead to a state called velopharyngeal insufficiency (VPI) and most common cause of this condition is cleft palate. Even after repair surgery, VPD has been found among cleft patients in range of 30~50 % [4].

Diagnosis of VPD, identifying a critical cause of the dysfunction, can be carried out through physical and oral examination, perceptual speech assessment, radiographic mulitplanar videofluoroscopy and nasendoscopy. Treatment options of VPD with history of cleft palate repair include surgical and prosthetic interventions in combination with speech therapy. Various surgical techniques, such as pharyngeal flap surgery, sphincter pharyngoplasty and Furlow palatoplasty, have been used, but success rate of the surgical treatment is approximately 50 % [5, 6]. Prosthetic devices for VPD can be alternative treatment method when surgical approach is not considered. Widely used types of these devices, called speech aids, are palatal lift appliance and speech bulb.

The purpose of this report was to introduce an unusual case of VPD that was successfully treated using a prosthetic device and speech therapy and to increase an attention on the effectiveness of speech aid prosthesis.

## Case presentation

A 16 year old female had suffered from nasal sound during conversation, and she was very depressed and stressful due to her condition. The patient had a history of incomplete cleft palate that was repaired 15 years ago. According to the patient, she had received speech therapy at local clinic several times, but the patient believed that there was no obvious change after the therapy. Clinical examination revealed that she had seemingly insufficient palatopharyngeal tissue. Speech and voice assessment was conducted. Speech resonance was measured using nasometer (Nasometer II model 6200-3, Kay Elementrics Corp., USA) which can calculate a ratio of the acoustic energy collected by the two separate microphones placed near to nose and mouth. This ratio means nasalance and higher percentage indicates higher nasality. Simple vowels (/a/, /i/, /e/, /o/, /u/), diphthong (/ja/, /je/, /wi/) and two passages were repeated for the test. As a result, the vowel /i/ revealed severe nasalance (78.4 %, mean value of 22.3 %) and high nasalance was found on /u/, /je/. Syllable repetition test indicated a hyper-nasality on oral consonants (Table 1). Articulation differential test showed relatively high percentage of correct consonants (97 %); however, a distortion of a specific consonant /s/ was found. Maximum phonation time (MPT), sustaining phonation of a vowel sound /a/ as longer as patient can do, was only 9.3 second which was short for her age representing an air escape thorough a nasal pathway. Overall assessment indicated that minor velopharyngeal insufficiency with the pattern of phoneme-specific nasal emission. Bulb type prosthesis with intensive speech therapy was planned.

**Table 1:** Results of nasometric assessment before and after intervention

	Nasalance score (%)							
Vowels	/a/	/i/	/e/	/o/	/u/	/ja/	/je/	/wi/
Mean (%)	8.6	22.3	8.7	8.4	10.0	8.5	8.6	20.5
Before Intervention	37.5	78.4	49.5	42.3	53.4	35.5	53.9	39.2
After Intervention	4.1	12.7	3.3	2.7	4.3	3.7	5.7	9.5

**Fig. 1:**
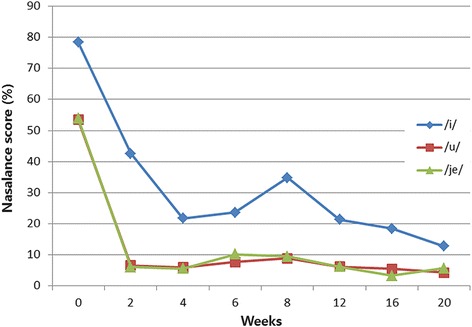
Changes of nasalance score for vowels before and after placement of the speech aids

A careful impression with adequate extension to the soft palate was taken. Then, the palatal portion of speech aid with posterior wire extension was fabricated by acrylic resin. The appliance was delivered to the patient and initially she complained of gaging reflex. After 2 weeks of adaptation period, pharyngeal portion was shaped using high-viscosity impression wax during production of oral pressure sounds which cause velopharyngeal function. Modification of the bulb was continued until remarkable reduction in nasal emission was observed. This pharyngeal portion of the speech aid was replicated in acrylic resin. The patient was recommended to have speech therapy once a week at least. After the delivery of the appliance, the nasometric assessment was conducted by 2, 4, 6, 8, 12, 16, 20 weeks, and the result was recorded (Fig. 1). The nasalance score was dramatically reduced in two weeks and the score was consistently sustained. Wearing time reduction and speech bulb reduction was carried out by 12 and 16 weeks follow-up session respectively. After 20 weeks of follow-up, she eventually did not want to wear the appliance any longer since the annoying problem of nasal voice was disappeared.

## Discussion

Speech prosthesis can be used when the surgical approach is contraindicated, systemic training of velopharyngeal function is necessary, or effect of improved velopharyngeal closure on the speech is to be evaluated [6]. And objective methods to determine the treatment option, either surgery or conservative intervention for VPD, was discussed by Shin (Table 2). According to the Shin’s criteria a severe nasalance score over 60 % should be recommended for the surgical intervention for VPD [7]. However, the patient in this report showed a severe hyper-nasality on specific vowel /i/ of 78 % and the nasalance was successfully decreased after using the prosthesis. The patient also showed selective nasal emission that affected production of certain high-pressure consonants /s/ while the other consonants were spoken in normal range. This pattern of nasalance is not common in the cleft patients with persisting postoperative nasal emission that is not restricted to a certain sound group but is rather pervasive [8]. Furthermore, the nasalance score was consistently decreased during the reduction program period. The time reduction started only 2 months after using the appliance and bulb reduction was carried out twice. Due to the consistent and stable decrease pattern of the nasalance, patient can take off the appliance 5 months after the initial delivery which was relatively short period of time: duration of using speech bulb is typically more than a year [9].

**Table 2:** Degree of nasalance and suggested treatment options for VPD (Shin’s criteria)

Nasalance		Recommended options of treatment
Below 20 %	No nasality	
20 ~ 35 %	Mild nasality	Speech therapy
35 ~ 45 %	Moderate nasality (marginal VPD)	Speech aid appliance with speech therapy
45 ~ 60 %	High nasality	Surgery or speech aid
Over 60 %	Severe nasality (VPD)	Surgery

A rational explanation of this successful outcome of the presented case was not fully understood. In this case, specific consonant /s/ was only one with distortion which was affected by nasal emission. And vowel /i/ was also showed prominent and severe nasalance comparing the other vowels. Because of this specificity, fabrication of speech aid and speech therapy could be focused on the distinct target that should be corrected. And because of a psychological stress on her nasal sound, the patient was very cooperative with long time wearing of the appliance and intensive speech therapy.

Speech therapy combined with the prosthesis is widely accepted treatment method for VPD. However due to the low predictability, some clinicians may be negative to this treatment method. According the several literature, successful outcome of the therapy were reported as approximately around 10~30 % [10–12]. However, there is still a possibility of improvement in this poor success rate. According to Yamashita et al., success rate, a percentage of patients who finally removed the prosthesis, was approximately 40 % when the therapy was applied on children less than 7 years old [9]. With consideration of commonly poor treatment compliance of children, this rate was quite high and it suggests that early intensive treatment could improve the prognosis.

Furthermore, using speech aid can avoid permanent complications of surgical intervention such as snoring, sleep apnea, airway obstruction and hyponasality [5, 13]. For the case of delayed surgery, temporary use of the prosthesis can train the velopharyngeal function and minimize the speech mislearning – the effectiveness of pre-operative prosthesis therapy already have reported [14]. Therefore, ahead of surgical intervention, speech therapy with the prosthesis should be considered as early as possible. And, all of the cleft team members who take care of VPD should make a greater effect to improve the compliance of the young patients and their parents.

## Conclusion

This clinical report introduces a case of VPD which was successfully and promptly treated with speech therapy using the speech aid. This effective intervention may be due to the fact that nasal emission affected only single consonant /s/ and this phonemic target can be focused well during the therapy. This report also suggests that pre-operative use of the prosthesis even in the patient group of severe nasal score, since young children showed better outcome and early use of the speech aid can improve the prognosis of the velopharyngeal surgery.

## Consent

Written informed consent was obtained from the patient for the publication of this report and any accompanying images.

## Abbreviations

VP: Velopharyngeal
VPD: Velopharyngeal dysfunction
VPI: Velopharyngeal incompetence; velopharyngeal insufficiency
